# Complete Genome of Rose Myrtle, *Rhodomyrtus tomentosa*, and Its Population Genetics in Thai Peninsula

**DOI:** 10.3390/plants12081582

**Published:** 2023-04-07

**Authors:** Matsapume Detcharoen, Sara Bumrungsri, Supayang Piyawan Voravuthikunchai

**Affiliations:** 1 Division of Biological Science, Faculty of Science, Prince of Songkla University, Hat Yai 90110, Thailand; 2 Center of Antimicrobial Biomaterial Innovation-Southeast Asia, Faculty of Science, Prince of Songkla University, Hat Yai 90110, Thailand; 3 Natural Product Research Center of Excellence, Faculty of Science, Prince of Songkla University, Hat Yai 90110, Thailand

**Keywords:** Ceylon hill gooseberry, ISSR, microsatellite, Myrtaceae, Myrtales, *Rhodamnia argentea*, rose myrtle

## Abstract

Several parts of rose myrtle, *Rhodomyrtus tomentosa*, exhibited profound antibacterial and anti-inflammatory activities, suggesting its potential in healthcare and cosmetics applications. During the past few years, the demand for biologically active compounds in the industrial sectors increased. Therefore, gathering comprehensive information on all aspects of this plant species is essential. Here, the genome sequencing using short and long reads was used to understand the genome biology of *R. tomentosa*. Inter-simple sequence repeats (ISSR) and simple sequence repeats (SSR) markers, and geometric morphometrics of the leaves of *R. tomentosa* collected across Thai Peninsula, were determined for population differentiation analysis. The genome size of *R. tomentosa* was 442 Mb, and the divergence time between *R. tomentosa* and *Rhodamnia argentea*, the white myrtle of eastern Australia, was around 15 million years. No population structure was observed between *R. tomentosa* on the eastern and western sides of the Thai Peninsula using the ISSR and SSR markers. However, significant differences in leaf size and shape of *R. tomentosa* were observed in all locations.

## 1. Introduction

Rose myrtle, *Rhodomyrtus tomentosa* (Aiton) Hassk., is an evergreen flowering plant belonging to the order Myrtales of the family Myrtaceae. This plant species is native to Southeast Asia and is commonly found far and wide from South China to Indonesia [1]. In Thailand, *R. tomentosa* can be found on the east and west sides of the Malay Peninsula (Thai Peninsula) [2]. It thrives in poor acidic soils, especially near the coastlines and sunny environment [2,3,4]. Bees are the primary pollinators of *R. tomentosa* as this plant species cannot be pollinated by wind [5]. Several parts of this species are rich in sugar, vitamins, and minerals. Most parts of the plants have been used in traditional medicine. Leaves have been used to treat colic diarrhea [6], abscesses, hemorrhage [7], and unripe fruits for treating sepsis, colic, and diarrhea [6,8].

Numerous compounds of *R. tomentosa* have been investigated to search for alternative active compounds, given their detrimental effects against several symptoms. Rhodomyrtone, an acylphloroglucinol found in the leaves of *R. tomentosa*, exhibited diverse effects, such as antibacterial, anti-inflammatory, antioxidant, and antitumor activities [9,10,11,12]. It is used in cosmetics and therapeutics, such as acne creams [13]. Similarly, piceatannol is a primary phenolic compound found in this plant, possessing antioxidant, anti-cancer, and anti-inflammatory effects [14].

Myrtaceae family encompasses approximately 150 genera and 5000 species, with a genome size varying from 187 Mb in *Syzygium oleosum* to 962 Mb in *Eucalyptus rudis*. Despite the presence of several species in this family, genomic studies on Myrtaceae have mainly focused on *Eucalyptus* species [15]. In *R. tomentosa*, the chloroplast genome was approximately 156 kb in size [16], while the genome of *R. tomentosa* cultivar LFSTJN1 in China was around 470 Mb [17]. The cytological study reported that diploid cells contained 22 chromosomes [18]. Furthermore, inter-simple sequence repeats (ISSR) and simple sequence repeats (SSR) have been employed to examine the population genetics of *R. tomentosa* in Malaysia and China, respectively [19,20]. However, more information is needed on this species’ population genetics and genetic diversity in other geographical regions [19].

Herein, we aimed to understand the genome biology, evolutionary history, and genetic diversity of *R. tomentosa.* Despite abundant knowledge of *R*. *tomentosa* organic compounds, evolution, and genome biology, its genetic diversity must be better known than other Myrtaceae members. The genome of *R. tomentosa* was sequenced and assembled through short and long reads, and the genetic diversity within the Thai Peninsula was detected using ISSR and SSR markers. We found that the genome of *R. tomentosa* was similar to those of other Myrtaceae members. However, no significant genetic differentiation was observed across *R. tomentosa* in the Thai Peninsula.

## 2. Results

### 2.1. Genome Assembly

The estimated genome size of *R. tomentosa* was between 442 and 485 Mb. The assembled genome of *R. tomentosa* was sequenced using short- (45.1× coverage) and long-read sequencing (12.1× coverage). After removing contaminant and chloroplast reads (Figure S1), the final length of the assembly was 442 Mb, with a GC content of 40.5%, 1431 contigs, and N50 of 868,979 bases (Table S1, BioProject accession PRJNA846150). The completeness of the assembly determined by BUSCO against the eudicots odb10 database of 2326 genes was 94.8% (2205 genes), of which 92.7% (2156 genes) were complete and single copies, 2.1% (49 genes) were complete and in duplicates, 1% (23 genes) was fragmented, and 4.2% (98 genes) were missing. Repeat analysis revealed that Gypsy/DIRS1 long terminal repeat retrotransposons were the most dominant (Table S2). *Rhodomyrtus tomentosa* contains more retroelements yet fewer DNA transposons and small RNAs than *R. argentea*.

**Figure 1:**
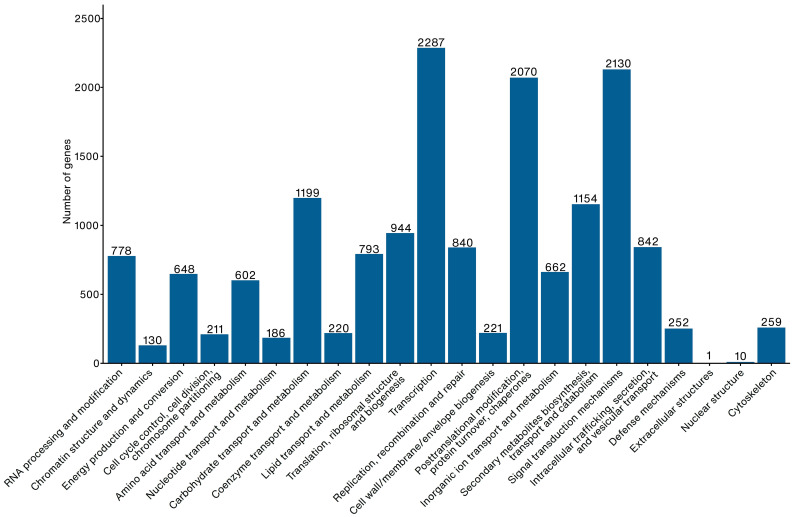
Gene function of *Rhodomyrtus tomentosa* classified based on clusters of orthologous groups database.

Gene prediction identified 31,779 transcripts of 29,768 predicted genes. Five mean exons and four mean introns were observed per transcript, respectively. The mean gene length was 3157 bases. The longest coding sequence identified was 16,374 bases, with the closest match predicted to the midasin gene of *R. argentea* (XM_030670374.1) (Table S3). BUSCO completeness analysis of protein-coding sequences against the eudicots odb10 database reported completeness of 92.2%, of which 80.7% were complete and single copies, 11.5% were complete and in duplicate copies, 2.1% were partially complete, and 5.7% were missing. About 28,375 genes were annotated for gene annotation with most having an unknown function, cytochrome P450, protein kinases, and UDP-glycosyltransferase family (Table S4). Furthermore, KEGG pathway analysis indicated that most annotated genes were associated with secondary metabolites’ metabolic pathways and biosynthesis (Table S5). The functional classification of the genes showed that most *R. tomentosa* genes belong to transcription, signal transduction mechanisms, post-translational modification, protein turnover, and chaperone functions (Figure 1).

For orthologous gene analysis, approximately 96% (704,329 genes) of genes present in all thirteen Myrtaceae species were assigned to 42,372 orthogroups. Of these orthogroups, 9858 existed in all species. In the case of *R. tomentosa*, 84% of its genes were assigned to 15,587 orthogroups. Among these, 833 orthogroups, representing 2308 genes, were specific to *R. tomentosa* (Table S6).

**Figure 2:**
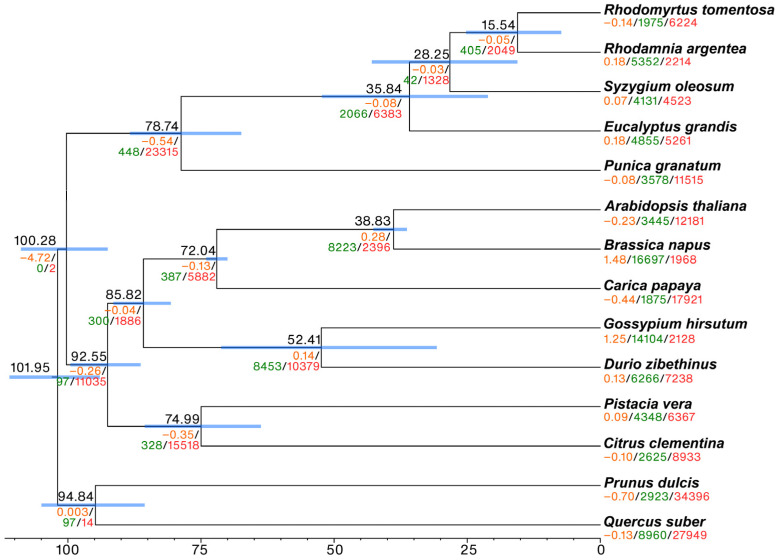
Gene family evolution conducted on clock-calibrated Bayesian phylogenetic tree based on orthologous genes. Average expansion rate, number of genes expanded, and number of genes contracted are shown in orange, green, and red, respectively. Mean and 95% confidence interval of the estimated divergence time (million years ago) are shown above each node and blue horizontal bar, respectively.

Based on 236 nuclear orthologous gene groups (Figure S2) or chloroplast genomes (Figure S3), the maximum likelihood phylogenetic trees had similar topologies. Myrtaceae species, along with *R. tomentosa*, formed a monophyletic clade. The clock-calibrated phylogenetic tree reported that the split between the closest taxa, *R. tomentosa* and *R. argentea*, occurred around 15.5 (7.3–25.2, 95% confidence interval) million years ago (Figure 2).

The significance of gene family evolution across Myrtaceae species was based on the clock-calibrated phylogenetic tree. Overall, a negative rate of gene expansion/contraction (−0.05) was observed in *R. tomentosa* (Figure 2). A total of 352 gene families with significant expansion or contraction rates were detected in *R. tomentosa.* Numerous genes within these families belong to protein kinases, leucine-rich repeats, and disease-resistance proteins (Table S7).

**Figure 3:**
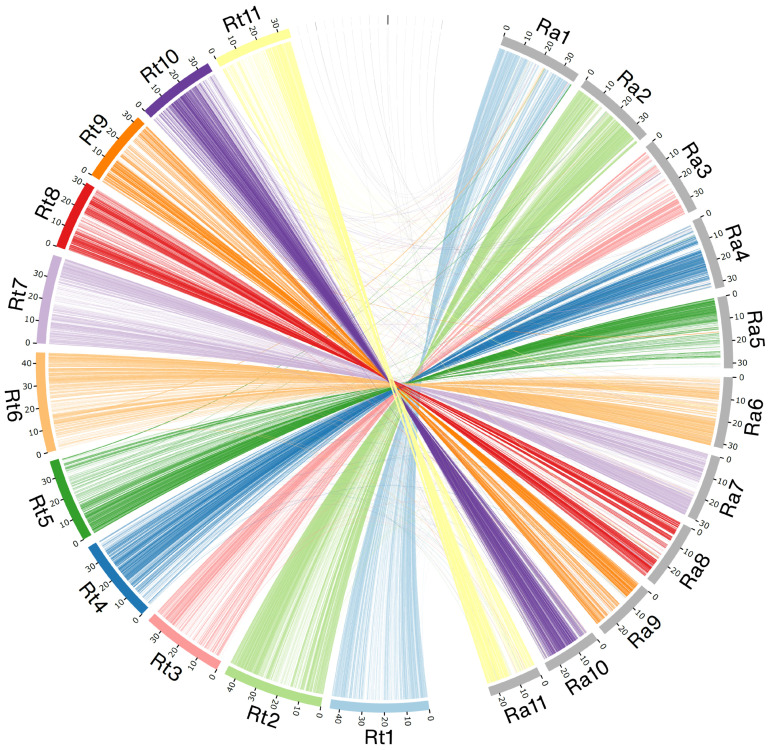
Genome synteny between *Rhodomyrtus tomentosa* pseudomolecules (Rt; colored) and *Rhodamnia argentea* chromosomes (Ra; gray). Contigs of *R. tomentosa* that are not able to scaffold into chromosomes are shown on top of the figure.

Comparing genome synteny between *R. tomentosa* and *R. argentea*, we found a strong synteny across the genomes of the two species (Figure 3). All pseudomolecules of *R. tomentosa* aligned well with the chromosomes of *R. argentea*. However, some synteny segments were on different chromosomes of *R. argentea*, particularly pseudomolecules 10 in *R. tomentosa*.

The terpene synthase genes of *R. tomentosa* and other Myrtaceae were examined. There were around sixty candidate terpene synthase genes in *R. tomentosa* (Table S4), and several contained all three domains (Table S8). The conserved motifs in these genes were organized similarly in *R*. *tomentosa*, *R. argentea*, and *E. grandis* (Figure S4). The phylogenetic analysis demonstrated that many *R. tomentosa* terpene synthase genes were similar to those of *Eucalyptus grandis*, while those of *Prunus dulcis* and *Punica granatum* formed different clades (Figure S5).

### 2.2. Population Genetics and Leaf Morphometrics of R. tomentosa on Thai Peninsula

Two markers, ISSR and SSR, were employed for population genetic analysis of *R. tomentosa* collected from twelve locations on the east and west sides of the Thai Peninsula (*n* = 80). For the ISSR marker, the mean expected heterozygosity per locus was 0.86. The ISSR primer, UBC825, had the highest expected heterozygosity and primer 56 had the most polymorphism information content (Table S9). Across the ten loci, we observed the highest expected heterozygosity in Phatthalung and Nakon-si-thammarat, and the lowest in Phuket (Table S10). The summary statistics of the SSR marker were similar to those of the ISSR marker. The expected heterozygosity per location ranged between 0.63 (Pattani) and 0.85 (Chumphon) (Table S10).

**Figure 4:**
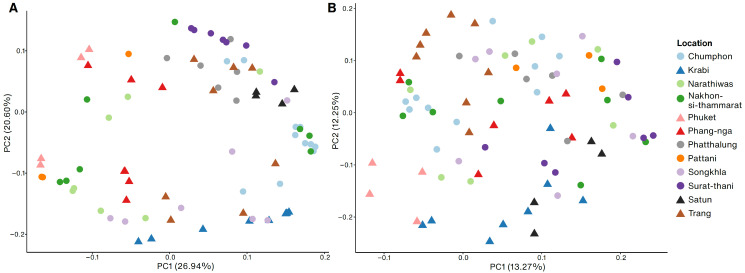
Principal component analysis based on ten ISSR loci (**A**) and thirteen SSR loci (**B**). Samples from the western and eastern side of Thai Peninsula are shown in triangles and circles, respectively.

We used principal component analysis (PCA) using the Lynch distance to observe similarity among the samples by ISSR or SSR marker. PCA revealed that the samples from the east and west sides of the Peninsula did not form distinct clusters (Figure 4). However, compared to using the SSR marker, the PCA of the ISSR markers showed more grouping of samples within the location, for example, samples from Krabi, Satun, and Surat-thani (Figure 4A). In addition, admixture analysis of the ISSR and SSR markers revealed two distinct genetic clusters (*K* = 2), with no differentiation between the east and west sides of the Peninsula (Figure S6). Notably, Chumphon, Songkhla, and Krabi exhibited high similarity.

**Figure 5:**
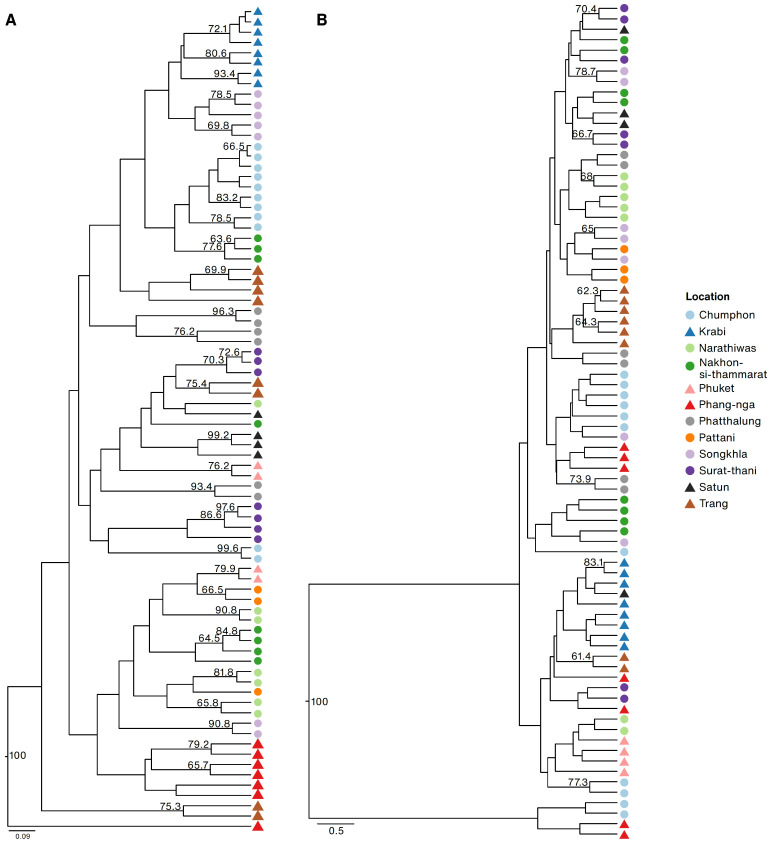
UPGMA dendrograms based on Nei’s genetic distance of *Rhodomyrtus tomentosa* samples collected from twelve locations in Thai Peninsula, using ISSR (**A**) and SSR markers (**B**). Bootstrap values higher than sixty are shown. Samples from the western and eastern side of the Thai Peninsula are shown in triangles and circles, respectively.

The UPGMA dendrogram based on the genetic distance of the ISSR marker showed more similarity across the samples within locations than using the SSR marker (Figure 5). The ISSR dendrogram was categorized into three clades. The first consisted of Phang-nga and Narathiwas; Surat-thani samples were placed in the second clade, and samples from Krabi, Songkhla, and Chumphon comprised the third clade.

The SSR marker provided more information about *G*_ST_ than the ISSR markers for pairwise population differentiation. Population differentiation based on ISSR and SSR markers revealed the highest *G*_ST_ values between Phuket and Chumphon and Phuket and Pattani, respectively (Table S11).

An evaluation of population differentiation between the east and west sides of the Peninsula was conducted using an analysis of molecular variance. For the ISSR marker, there was no significant difference between the east and west sides (AMOVA: *p* = 0.49). The variation among samples within locations between the sides was 32.39%, and the differentiation between the two sides was 0.32. For the SSR marker, there was no difference between the two sides (AMOVA: *p* = 0.07), and the variation and differentiation among samples within locations between the two sides were 16.05% and 0.16, respectively.

**Figure 6:**
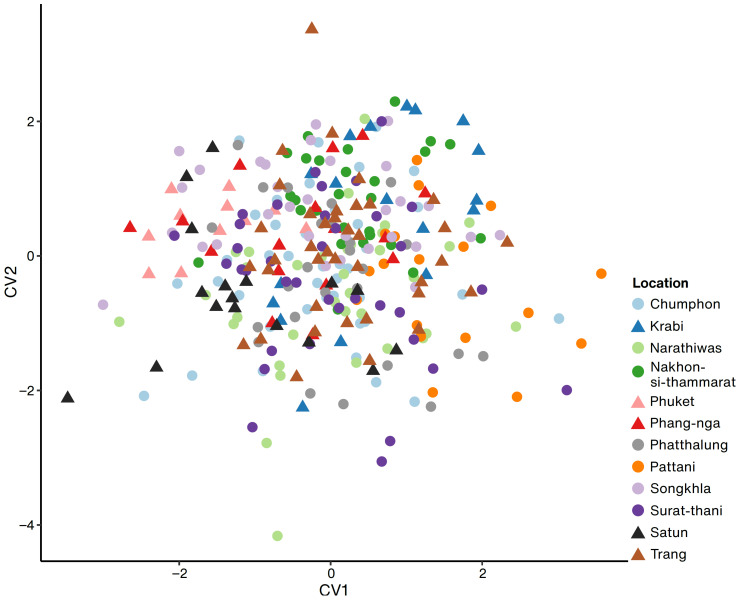
Canonical variate analysis of leaf geometric morphometrics across the twelve locations based on four landmarks. Populations in the western and eastern side of Thai Peninsula are shown in triangles and circles respectively.

Geometric morphometrics with four landmarks was used to determine whether there was a differentiation in leaf morphology among the locations. Leaf size and shape significantly varied across the locations (Procrustes ANOVA: size *F*_11,300_ = 5.10, *p* < 0.01 and shape *F*_44,1200_ = 4.49, *p* < 0.01). The longest Mahalanobis distance was observed between Phuket and Pattani, and the shortest was between Chumphon and Surat-thani (Table S12). We observed a high variation in the size and shape of leaves but no specific patterns based on the location or the east and west sides (Figure 6). In addition, Mantel tests were conducted to check the correlation between Nei’s genetic distance and Mahalanobis distance of morphometrics data. The results indicated no significant correlation between genetic distances for ISSR (*p* = 0.42), SSR markers (*p* = 0.51), and morphometric distances.

## 3. Discussion

### 3.1. R. tomentosa Genome

Herein, we reported the genome assembly of *R. tomentosa*. In our study, the genome size of *R. tomentosa* (442 Mb) was similar to the cultivar LFSTJN-1 in China (470 Mb) [17]. It was found to be approximately half the size of its sister species, *Rhodomyrtus psidioides* (BioSample SAMN19602563), with a genome size of 931 Mb. The differences in genome size can be attributed to the polyphyletic nature of *Rhodomyrtus*. *Rhodomyrtus tomentosa* and *R. psidioides* were placed in different clades based on the nuclear internal transcribed spacer [21]. The genome size variation is common within Myrtaceae, for example, in *Eucalyptus* [22,23]. Nevertheless, the genome size of *R. tomentosa* is in the same range as those of other Myrtaceae members (i.e., between 187 (*Syzygium oleosum*) and 962 Mb (*Eucalyptus rudis*)).

For gene family evolution, many genes related to disease resistance (e.g., code for tobacco mosaic virus resistance protein N and disease-resistance nucleotide-binding site–leucine-rich repeat family) were significantly expanded or contracted, indicating the reduced susceptibility of the plant species against pests and diseases [24]. The proteins of these genes provide protection against plant pathogens, including fungi [25,26], insects [27], bacteria [28], and viruses [29], in numerous plants.

We compared the genome synteny of *R. tomentosa* with *R. argentea* as they belong to the Myrteae tribe and have the same chromosome numbers [18]. We found that *R. tomentosa* demonstrated high collinearity with *R. argentea*, supported by their recent divergence time (7.3–25.2 million years). *Rhodomyrtus* and *Rhodamnia* are closely related, share similar evolutionary histories, are distributed in the same area (Southeast Asia, Australia, and Pacific islands), and produce similar organic compounds [30,31]. *Rhodomyrtus tomentosa* has twice as many Gypsy elements as *R. argentea*. In addition, *Rhodomyrtus tomentosa* has more repetitive elements than most Myrtaceae species, including *R. argentea*, *Psidium guajava*, *Corymbia citriodora*, and *Metrosideros polymorpha* [32,33,34]. The high accumulation of Gypsy in the *R. tomentosa* genome suggested a specific activity of the long terminal repeats in this species.

The estimated divergence period of Myrtales in the late Cretaceous was in line with estimations of previous studies based on nuclear and plastid sequences, consolidating orthologous genes from publicly available genome data [35,36]. The divergence time of *R. tomentosa* and other Myrtaceae occurred in the late Miocene, between 7.3 and 25.2 million years ago. This result was in line with a previous study that estimated the divergence time of *Rhodomyrtus* around 14.64–24.64 million years ago using pollen fossil, nuclear, and chloroplast sequences [31].

Myrtaceae are well-known for their terpene diversity [37]. The number of terpene synthase genes in Myrtaceae varies between species, from 37 in *Melaleuca alternifolia* [38] to 113 in *E. grandis* [39]. In *R. tomentosa*, around 60 putative terpene synthase genes were identified and the structures of the conserved motifs of these genes were similar to those in other species analyzed. Heterogeneity in secondary metabolite gene numbers reflects evolutionary divergences among species driven by biotic and abiotic factors, including herbivory and temperature [37,40]. Phylogenetic analysis confirmed that the terpene synthase genes of *R. tomentosa* were phylogenetically related to other Myrtaceae than Lythraceae (*P. granatum*) and Rosaceae (*P. dulcis*). Several *R. tomentosa* terpene synthase genes were clustered with germacrene D synthase genes, whose product, germacrene D, is one of the common volatile compounds found in plants, including Myrtaceae [37] that can be used against insects [41].

Compared to the findings reported by a previous study of the *R. tomentosa* cultivar in China [17], our study reported similar results, including gene count, mean exon per gene, percentage of repeats in the genome, and predicted divergence time. These findings contributed to a more comprehensive understanding of the genomics of *R. tomentosa* and its evolutionary relationships within the Myrtaceae family.

### 3.2. Population Genetics and Geometric Morphometrics of R. tomentosa in Thai Peninsula

Comparative analysis of ISSR and SSR markers has been used to evaluate genetic diversity in numerous plant species, including the Myrtaceae family [42,43]. For this study, we used the ISSR and the SSR markers to study the population genetics of *R. tomentosa*. Both markers exhibited similar basic statistics per locus, including genetic diversity and heterozygosity. However, the dendrogram based on genetic distance and PCA revealed that the ISSR delivered better resolution for sample classification than the SSR markers. In addition, population analysis of *R. tomentosa* using the markers revealed high expected heterozygosity in the Thai Peninsula, suggesting high genetic diversity in the area, which was considerably greater than that observed in *R. tomentosa* populations in Malaysia [19] and China [20].

The PCA, genetic distance dendrogram, and admixture analysis indicated a lack of differentiation between the two sides of the Peninsula, despite the presence of mountain ranges as geographic barriers. This suggested that gene flow occurred between these two sides. Another population genetics study of *R. tomentosa* within Peninsular Malaysia did not find genetic differentiation between the east and west sides [19]. Unlike mangroves and other marine plants [44,45,46], mountain ranges did not act as geographic barriers for *R. tomentosa*. Nevertheless, PCA showed similarities among samples from the exact location, suggesting some degree of inbreeding. For instance, both markers showed similarities between samples from Krabi, possibly due to close proximity of the *R. tomentosa* collected in this area compared with those from other locations.

Based on population differentiation analysis, low *G*_ST_ values were observed between nearby locations; for example, collection sites at Songkhla and Phatthalung were around thirty kilometers apart with no geographic barriers. The highest *G*_ST_ values were observed between Phuket–Chumphon and Phuket–Pattani. Phuket was the only non-mainland location in this study, making it challenging for gene flow between Phuket and other locations. Additionally, *R. tomentosa* has a geitonogamy mating system and can only be outcrossed by insect pollinators (e.g., bees) but cannot be pollinated by wind [5]. The effects of geographic barriers on island populations have been documented in several plants, such as *Trifolium repens* [47] and *Lactuca watsoniana* [48].

Most morphometrics studies in Myrtaceae have focused on variations in complex species, such as *Myrcia* [49], *Eugenia* [50], and *Eucalyptus* [51]. Less is known about population variations. Herein, we observed high variation in leaves within locations. The leaves collected from Phuket and Pattani had the highest Mahalanobis distance, similar to the results from population differentiation analysis using the SSR marker. The study of the morphological variation of another Myrtaceae, *Myrceugenia fernandeziana*, could not find any differentiation across populations [52]. However, adding more morphological characteristics, such as flowers and peduncles, might increase the resolution of classification [51].

In conclusion, the assembled genome of *R. tomentosa* can aid in implementing the genetics and evolutionary studies of other Myrtaceae members. Herein, we assembled the 442 Mbp genome of *R. tomentosa* using short- and long-read sequencing. High synteny was observed between the genomes of *R. tomentosa* and *R. argentea*. The population genetic analysis of *R. tomentosa* collected from twelve distinct locations across the Thai Peninsula using ISSR and SSR markers demonstrated congruent results. There was neither strong genetic structure nor genetic differentiation between the east and west sides of the Peninsula, suggesting that mountain ranges did not act as a strong geographic barrier against this plant species.

## 4. Materials and Methods

### 4.1. Genome Assembly

The leaves of *R. tomentosa* were collected from Hat Yai, Thailand (7°00′24.6″ N, 100°30′14.8″ E), in October 2021. The DNA was extracted from a single young leaf using the cetyltrimethyl ammonium bromide (CTAB) method [53] and purified with Mag-Bind TotalPure NGS (Omega Bio-tek, Norcross, GA, USA) by the manufacturer’s instructions. The final product was sent to BGI Genomics (Hong Kong, China) for 2 × 150 bp short-read sequencing. The raw reads were checked for quality using Fastp version 0.23.0 [54] with a minimum length of 30 and a sliding window of four with a mean quality of 30. To estimate the genome size of *R. tomentosa*, we used Jellyfish version 2.3.0 [55] based on the filtered short reads with k-mer lengths of 19, 21, and 31, and the results were plotted using GenomeScope version 2.0 [56].

For long-read sequencing, DNA library was prepared using the NEBNext Companion Module for Oxford Nanopore Technologies ligation sequencing (New England BioLabs, Ipswich, MA, USA) and ligation sequencing kit (LSK-SQK109; Oxford Nanopore Technologies, Oxford, UK). The library was sequenced on an R9.4.1 flow cell on a MinION device.

Guppy version 6.0.1 (Oxford Nanopore Technologies, Oxford, UK) was used for basecalling with a high accuracy option and a minimum score of 9. The reads were assembled using Flye version 2.9 [57] with a minimum overlap set to 3000. The assembled contigs were polished with four rounds of Racon version 1.5.0 [58] and one round using Medaka version 1.5.0 (Oxford Nanopore Technologies). Following this, the assembled contigs were polished with short reads using four rounds of Pilon version 1.24 [59]. Purge_Dups version 1.2.5 [60] was used to check the haplotypic duplication of the assembly. BlobToolKit version 3.1.0 [61] was used to check for contaminant reads, which were manually removed. Quast version 5.0.2 [62] and Benchmarking Universal Single-Copy Orthologs (BUSCO) version 5.3.0 [63] with the eudicots odb10 database were used to evaluate the genome assembly.

For repeated gene prediction, the consensus sequence was soft-masked using RepeatModeler version 2.0.2 [64] and RepeatMasker version 4.1.2 [65] with the *E. grandis* repeat library from the Plant Repeat Database [66]. The masked consensus sequence was aligned with the publicly available *R. tomentosa* transcriptome data (Biosample SAMN06640542) using rnaSPAdes version 3.15.4 [67]. The masked sequence, aligned transcriptome, and protein sequences of other Myrtaceae downloaded from UniProt [68] were used as inputs in AUGUSTUS version 3.4.0 [69] and GeneMark_ES version 4.69 [70] incorporated in Braker2 version 2.1.6 pipeline [71]. BUSCO version 5.3.0 [63] with the eudicots odb10 database was used to evaluate the genome assembly and repeat gene prediction. Gene ontology and KEGG (Kyoto Encyclopedia of Genes and Genomes) pathways of the predicted genes were performed using eggNOG-mapper version 2.1.7 [72].

OrthoFinder version 2.5.4 [73] was used to identify the orthologous genes of *R. tomentosa* by running all-versus-all diamond BLAST against thirteen other annotated eurosid genomes, namely, *Arabidopsis thaliana* (GCF000001735.4), *Brassica napus* (GCF000686985.2), *Citrus clementina* (GCF000493195.1), *Carica papaya* (GCF000150535.2), *Durio zibethinus* (GCF002303985.1), *E. grandis* (GCF016545825.1), *Gossypium hirsutum* (GCF007990345.1), *P. dulcis* (GCF902201215.1), *P. granatum* (GCF007655135.1), *Pistacia vera* (GCF008641045.1), *Quercus suber* (GCF002906115.1), *R. argentea* (GCF900635035.1), and *Syzygium oleosum* (GCF900635055.1). A maximum likelihood phylogenetic tree based on orthologous genes (Supplementary data S1) was constructed using FastTree version 2.1.11 [74] with local support values based on the Shimodaira-Hasegawa test, and others were left as default.

To estimate the divergence time of *R. tomentosa*, we used the MCMCTree program in PAML version 4.9j [75] with a phylogenetic tree based on orthologous genes and the concatenated translated nucleotide alignments of orthologues as inputs. The estimated divergence time of 37.4–49.5 million years between *Arabidopsis* and *Brassica* [76], 70–74 million years between *Arabidopsis* and *Carica* [77], and 66.4–112.6 million years between *Eucalyptus* and *Punica* [78] were used as calibration points. We performed a Markov chain Monte Carlo (MCMC) run for one million generations and a burn-in of 100,000 generations. with the JC69 model. Two independent MCMC runs were performed.

CAFE version 4.2.1 was used to analyze gene family evolution of *R. tomentosa* [79]. The orthologous gene counts from OrthoFinder and the clock-calibrated tree was used as inputs. A gene family was counted as a significant expansion or contraction when its p-value was less than 0.01.

Synteny analysis between *R. tomentosa* and *R. argentea* was compared. *Rhodamnia argentea* was selected as it is the closest species to *R. tomentosa* with a publicly available genome, and both species have eleven chromosomes [18]. The *R. tomentosa* assembled contigs were scaffolded into eleven pseudomolecules using the RagTag version 2.1.0 [80]. These pseudomolecules were mapped to the *R. argentea* reference genome (GCF_020921035.1) in a one-to-one relationship using the nucmer function implemented in MUMmer version 3.1 [81]. The alignment was filtered to retain only segments with more than 80 alignment identities with a minimum of 3000 bases. The lengths of the generated pseudomolecules were similar to those of Li et al. [17]. The synteny plot was generated using Circos version 0.69-8 [82].

The phylogenetic relationships and conserved motifs of terpene synthase genes of *R*. *tomentosa* were investigated. The terpene synthase amino acid sequences from *R. argentea*, *E. grandis*, *P. granatum*, and *P. dulcis* were downloaded from GenBank. The sequences were aligned using ClustalW version 2 [83]. A phylogenetic tree was constructed using IQ-TREE version 2.2.0 [84] with the GTR20+F+R6 model, 1000 approximate likelihood ratio tests, and 10,000 ultrafast bootstraps. The domains of terpene synthase, RRx8W, RDR, and DDXXD, of *R. tomentosa* were analyzed. The conserved motifs of the proteins of *R*. *tomentosa, R. argentea*, and *E. grandis* were analyzed using the Multiple Em for Motif Elucidation (MEME) program version 5.5.0 [85] with options set to any number of repetitions and the number of motifs was set to 10.

We mapped the long-read sequences to the published *R. tomentosa* chloroplast genome (NC_043848.1) using NOVOPlasty version 4.3.1 [86] to retrieve the chloroplast genome. For phylogenetic analysis, the chloroplast genomes were aligned with other plant chloroplast genomes, *Angophora floribunda* (NC_022411.1), *A. thaliana* (NC_000932.1), *B. napus* (NC_016734.1), *C. clementina* (NC_059803.1), *Corymbia maculata* (NC_022408.1), *C. papaya* (NC_010323.1), *Campomanesia xanthocarpa* (KY392760.1), *D. zibethinus* (NC_036829.1), *Eucalyptus camaldulensis* (NC_022398.1), *E. grandis* (NC_014570.1), *G. hirsutum* (NC_007944.1), *Pistacia vera* (NC_034998.1), *P. guajava* (NC_033355.1), *P. dulcis* (NC_034696.1), *P. granatum* (NC_035240.1), and *Syzygium samarangense* (NC_060657.1) using MAFFT version 7.505 [87] (Supplementary data S2). A maximum likelihood tree was obtained using the IQ-TREE version 2.2.0 [84] with the UNREST+FO+R3 model, 1000 approximate likelihood ratio tests, and 10,000 ultrafast bootstraps.

### 4.2. Population Genetic Analysis

**Figure 7:**
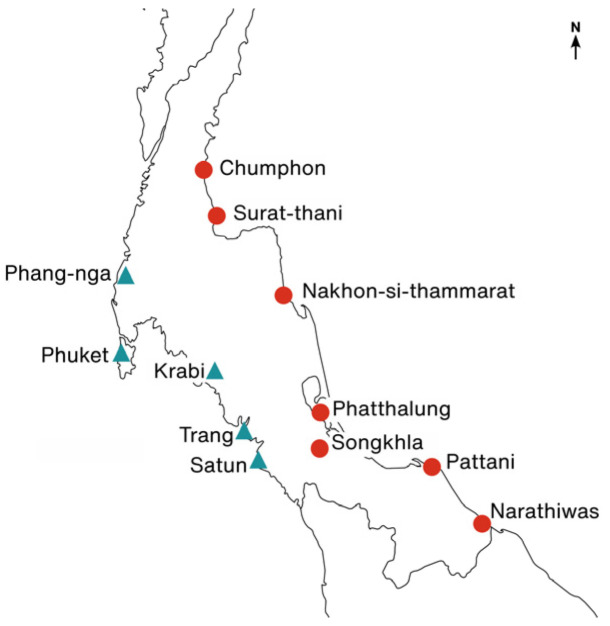
Twelve collection sites of *Rhodomyrtus tomentosa* across Thai Peninsula. Red circles and blue triangles denote the east and west sides of the Peninsula, respectively.

Leaves of *R. tomentosa* from twelve locations across the Thai Peninsula were collected in 2021 (Figure 7, Table 1). The leaves from three to eleven trees per location were collected as *R. tomentosa* was sparsely populated. The DNeasy Plant Mini Kit (QIAgen, Hilden, Germany) was used for DNA extraction. The DNA quality was analyzed with 1.5% agarose gel electrophoresis.

Population genetics was accessed using ISSR and SSR markers. Ten ISSR loci previously used by Hue et al. [19] to study the population genetics of *R. tomentosa* in Malaysia were selected. For the SSR marker, thirteen primers were designed by searching for sequences of di-, tri-, and tetranucleotide repeats in the *R. tomentosa* genome using WebSat [88] and Tandem Repeats Finder version 4.07b [89] (Table S13). All ISSR and SSR loci were amplified using AllTaq DNA polymerase (QIAgen, Hilden, Germany).

Fragment size scoring was performed using the R package Fragman version 1.09 [90] in R version 4.1.1 [91]. Simpson index, evenness index, expected heterozygosity, and analysis of molecular variance were calculated using the R package Poppr version 2.9.3 [92]. Mmod version 1.3.3 [93] was used to calculate Nei’s coefficient of gene differentiation (*G*_ST_) between the locations. The polymorphism information content of each ISSR and SSR locus was calculated using iMEC calculator [94]. Similarity among samples across populations was observed with PCA, calculated using Lynch distance using the R package adegenet version 2.1.6 [95]. The UPGMA dendrogram of the samples based on Nei’s genetic distance was constructed using Poppr version 2.9.3 [92] with 1000 bootstraps. The population structure was examined using Structure version 2.3.4 [96]. Five replicates were performed using a correlated allele frequency model for every 500,000 generations, with the number of genetic clusters (*K*) ranging from one to twelve. The first 50,000 burn-in iterations were discarded. The optimal number of populations was determined based on the highest Δ*K* value using Structure Harvester version 0.6.94 [97].

### 4.3. Geometric Morphometrics

The leaves of *R. tomentosa* from the twelve locations used in the population genetics analysis were used in geometric morphometrics. A total of 11–40 leaves per location were photographed. As the leaf venation patterns of *R. tomentosa* were challenging to observe, four landmarks were selected (Figure S7). A TPS file was created using tpsUtil64 version 1.81 (http://sbmorphometrics.org/soft-utility.html, 9 February 2023). Each leaf was digitized twice using the tpsDig2 version 2.31 (http://sbmorphometrics.org/soft-dataacq.html, 9 February 2023), and the mean landmarks of the two photographs were used for further analyses to reduce digitization errors. Procrustes analysis of variance of leaves among populations, regression, and canonical variate analysis was performed in MorphoJ version 1.07a [98]. Nei’s genetic distance of the ISSR and SSR markers and Mahalanobis distance of the geometric morphometrics data were used to test the correlation between genetic and morphometrics data. The correlation analysis was performed using the Mantel test function in vegan version 2.6-4 [99].

## Figures and Tables

**Table 1:** Collection sites, side of Thai Peninsula, and number of samples collected of *Rhodomyrtus tomentosa* for population genetic analysis.

Location	Coordinate	Side	n Samples
Chumphon	9°53′49.8″ N 99°08′16.2″ E	East	11
Krabi	7°51′43.4″ N 99°10′08.4″ E	West	8
Narathiwas	6°17′30.0″ N 101°58′41.3″ E	East	7
Nakhon-si-thammarat	8°37′09.7″ N 99°57′07.2″ E	East	8
Phuket	8°02′09.1″ N 98°17′51.5″ E	West	4
Phang-nga	8°49′36.1″ N 98°20′33.0″ E	West	7
Phatthalung	7°09′46.3″ N 100°04′58.4″ E	East	6
Pattani	6°52′10.4″ N 101°27′58.8″ E	East	3
Songkhla	7°13′45.0″ N 100°22′21.5″ E	East	7
Surat-thani	9°25′49.2″ N 99°16′16.2″ E	East	7
Satun	6°56′46.2″ N 99°41′44.5″ E	West	4
Trang	7°14′35.9″ N 99°33′01.4″ E	West	8

## Data Availability

All supplementary data, tables, and figures are available on OSF (DOI 10.17605/OSF.IO/ZHJDN, https://osf.io/zhjdn/ (9 February 2023)). Sequences of *R. tomentosa* are available in GenBank, BioProject accession number PRJNA846150.

## Supplementary Materials

The following supporting information can be downloaded at: https://www.mdpi.com/article/10.3390/plants12081582/s1, Figure S1: Blobplot of *Rhodomyrtus tomentosa* final assembly; Figure S2: Maximum likelihood phylogenetic tree based on 236 orthologous gene groups; Figure S3: Maximum likelihood tree based on chloroplast genome; Figure S4: Conserved motif analysis of terpene synthase genes in *Rhodomyrtus tomentosa*, *Rhodamnia argentea*, *Eucalyptus grandis*, *Punica granatum*, and *Prunus dulcis*; Figure S5: Phylogenetic tree of terpene synthase genes in *Rhodomyrtus tomentosa*, *Rhodamnia argentea*, *Eucalyptus grandis*, *Punica granatum*, and *Prunus dulcis*; Figure S6: Admixture analysis bar plots of genetic structure inferred by Bayesian clustering at *K* = 2 and 3 using ISSR and SSR data; Figure S7: Four geometric morphometrics landmarks used to digitized *Rhodomyrtus tomentosa* leaves; Table S1: Genome assembly statistics; Table S2: Repeats statistics of *Rhodomyrtus tomentosa* compared with *Rhodamnia argentea* genome; Table S3. Predicted genes statistics; Table S4: Gene ontology annotation of *Rhodomyrtus tomentosa* generated using eggNOG; Table S5. KEGG pathways identified from *Rhodomyrtus tomentosa* genes; Table S6: Statistics of orthologous gene analysis per species; Table S7: Orthogroups with significant expansion or contraction rate of *Rhodomyrtus tomentosa*; Table S8. Terpene synthase domains (RRx8W, RxR, and DDxxD) of *Rhodomyrtus tomentosa*; Table S9. Summary statistics per locus of ISSR and SSR markers; Table S10. Genotypic diversity per location calculated using ISSR and SSR markers; Table S11. Pairwise *G*_ST_ between *Rhodomyrtus tomentosa* locations based on ISSR and SSR markers; Table S12. Mahalanobis distances among *Rhodomyrtus tomentosa* populations; Table S13. Simple sequence repeats (SSR) loci developed from genomic data of *Rhodomyrtus tomentosa*.
