# Supplementary material for: Complete Genome of Rose Myrtle, Rhodomyrtus tomentosa, and Its Population Genetics in Thai Peninsula
Source: Plants (Basel). 2023 Apr 7;12(8):1582. doi: 10.3390/plants12081582 (PMC10144328; doi:10.3390/plants12081582)
Supplement: Supplementary file 1 [file plants-12-01582-s001.zip › SupplementFigures.pdf]

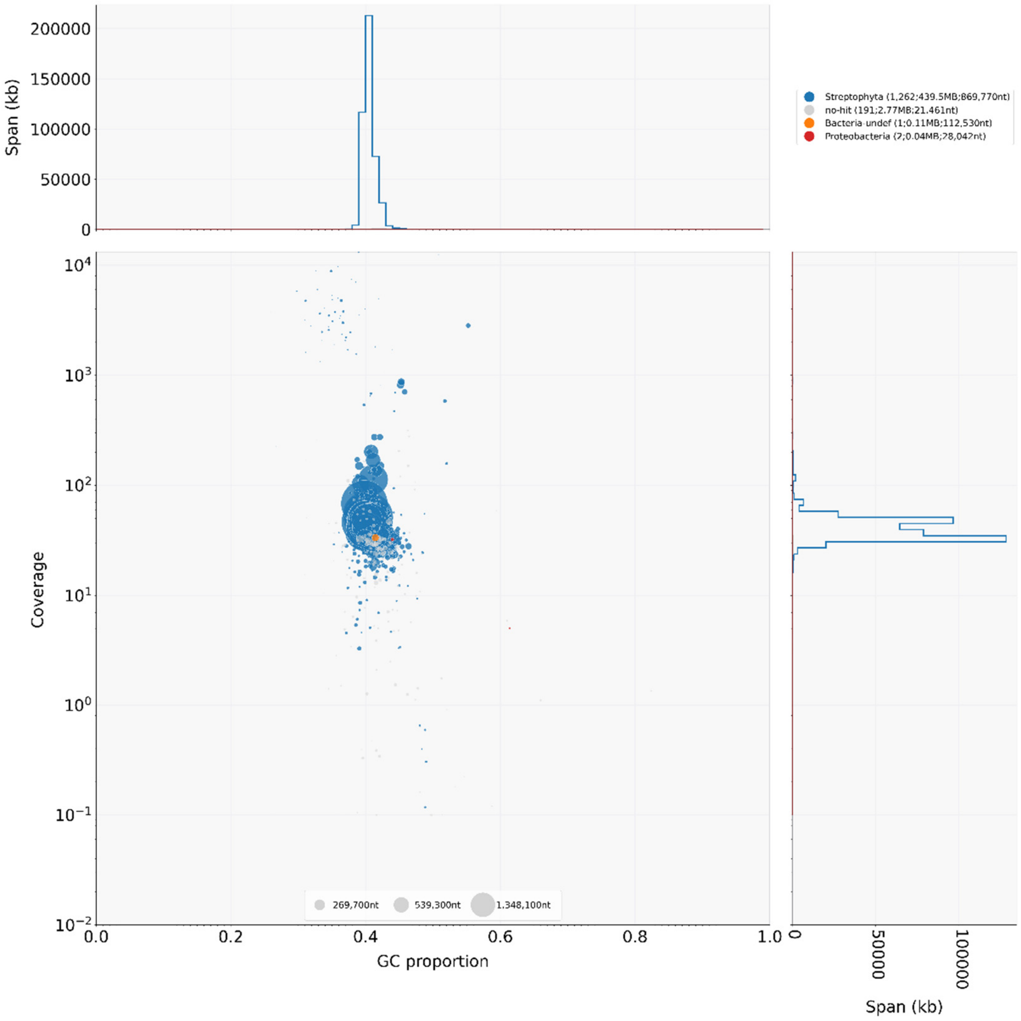

Figure S1. Blobplot of *Rhodomyrtus tomentosa* final assembly.

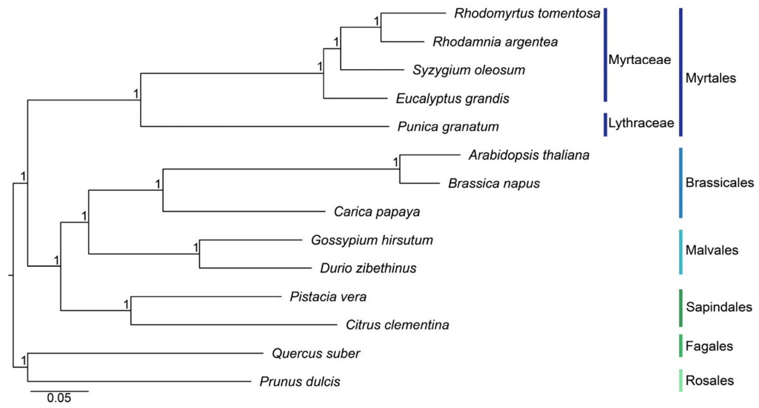

Figure S2. Maximum likelihood phylogenetic tree based on 236 orthologous gene groups. Local support values based on the Shimodaira-Hasegawa test are shown at each node.

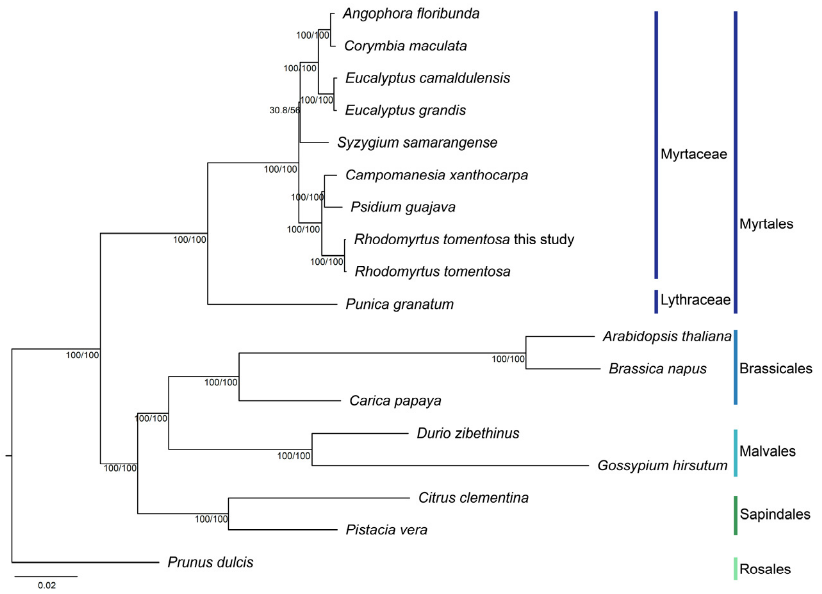

Figure S3. Maximum likelihood tree based on chloroplast genome. Approximate likelihood ratio test and rapid bootstrap numbers are shown at each node respectively.

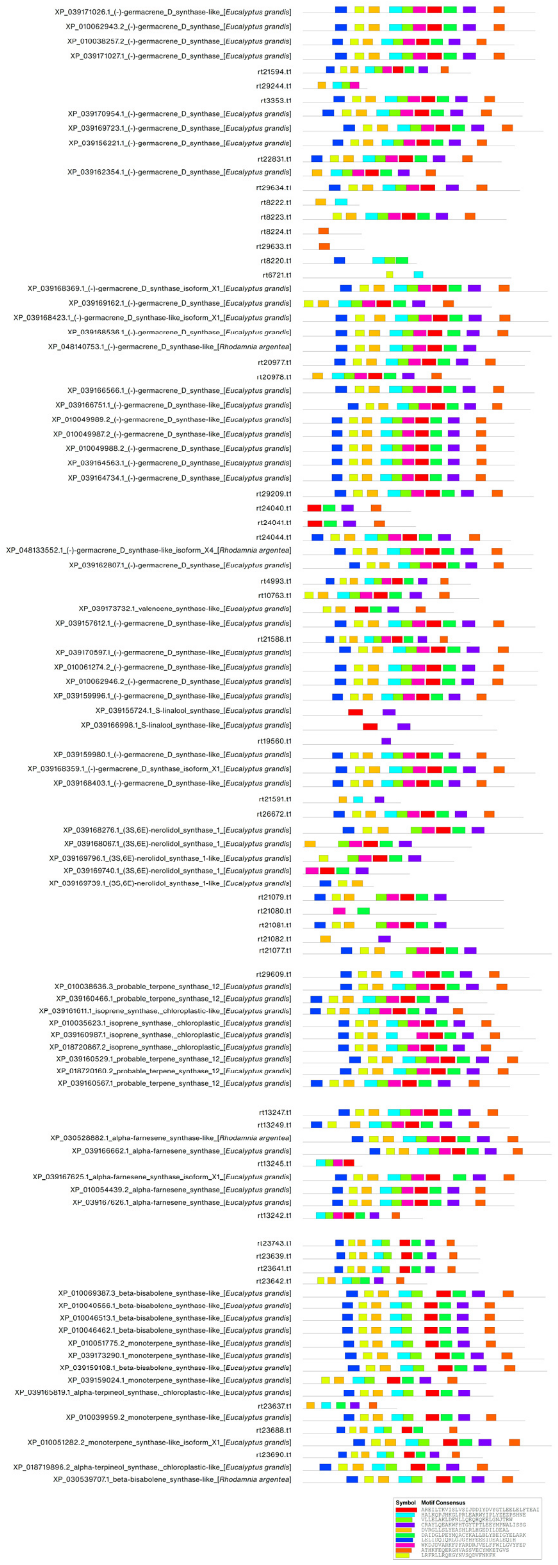

Figure S4. Conserved motif analysis of terpene synthase genes in *Rhodomyrtus tomentosa*, *Rhodamnia argentea*, *Eucalyptus grandis*, *Punica granatum*, and *Prunus dulcis*.

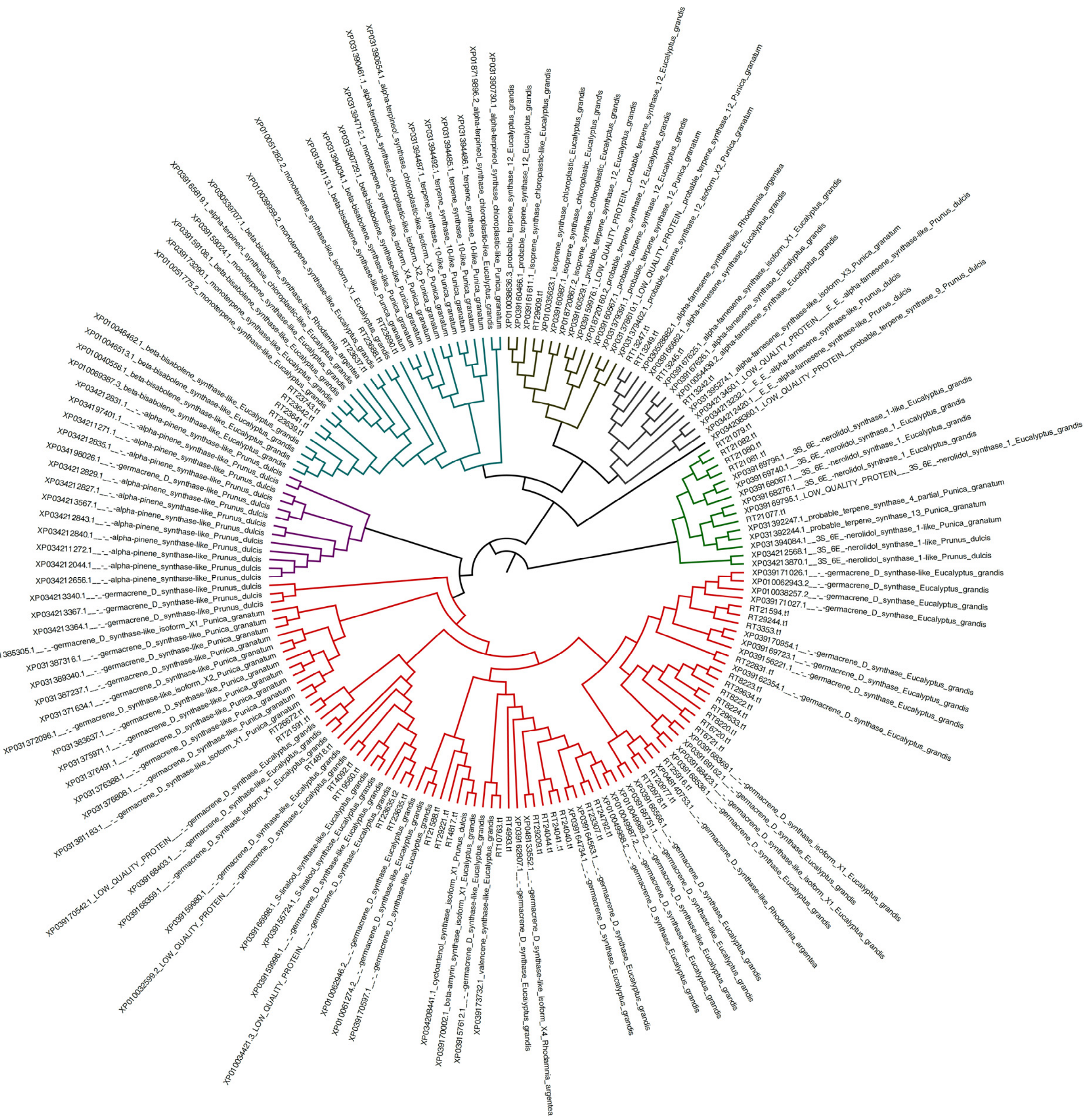

Figure S5. Phylogenetic tree of terpene synthase genes in *Rhodomyrtus tomentosa*, *Rhodamnia argentea*, *Eucalyptus grandis*, *Punica granatum*, and *Prunus dulcis*.

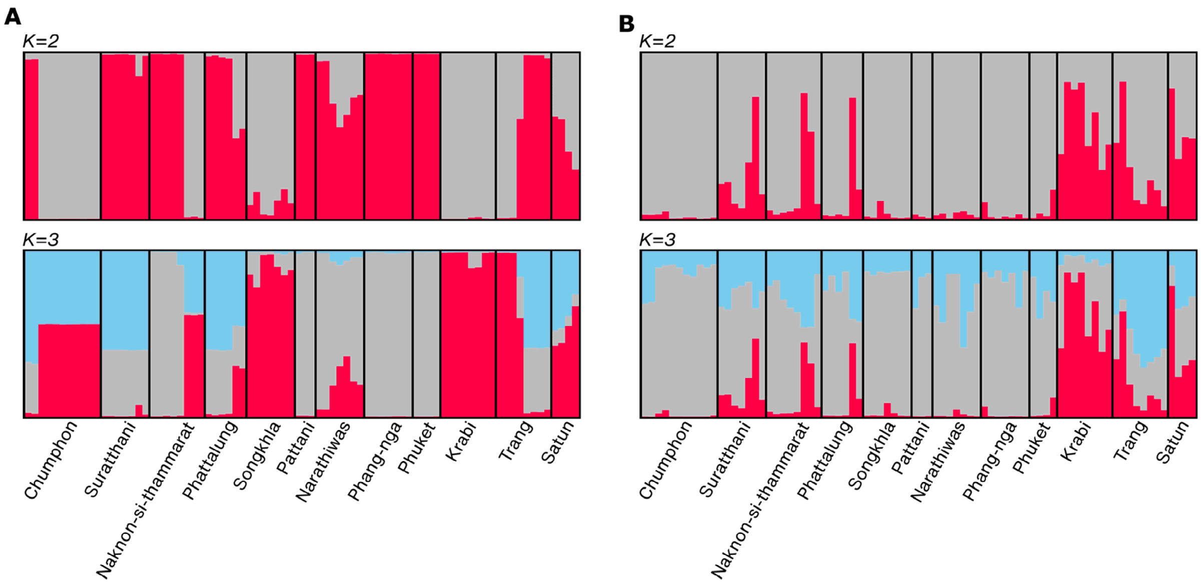

Figure S6. Admixture analysis bar plots of genetic structure inferred by Bayesian clustering at *K* = 2 and 3 using ISSR (A) and SSR data (B).

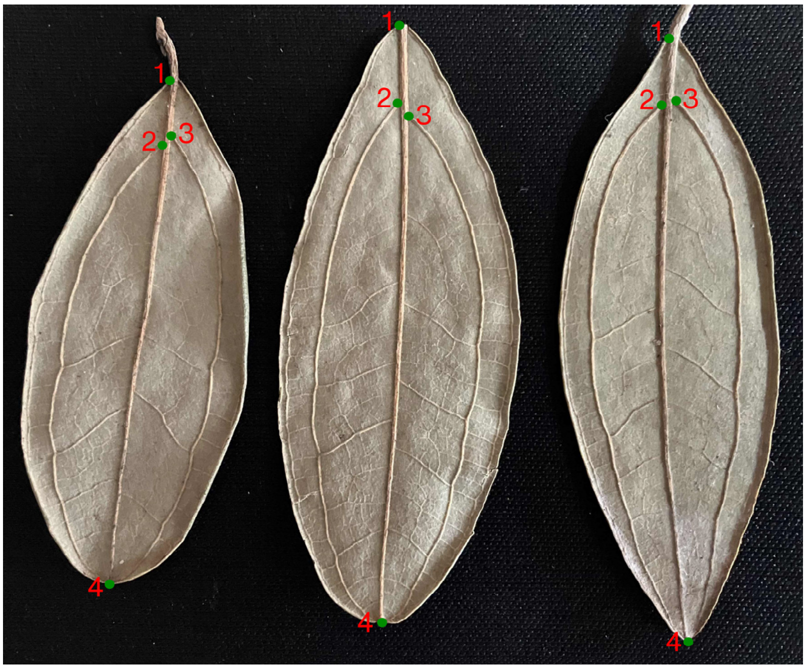

Figure S7. Four geometric morphometrics landmarks used to digitized *Rhodomyrtus tomentosa* leaves.
